# Mitochondrial genome variation of Atlantic cod

**DOI:** 10.1186/s13104-018-3506-3

**Published:** 2018-06-19

**Authors:** Tor Erik Jørgensen, Bård Ove Karlsen, Åse Emblem, Ragna Breines, Morten Andreassen, Trine B. Rounge, Alexander J. Nederbragt, Kjetill S. Jakobsen, Marianne Nymark, Anita Ursvik, Dag H. Coucheron, Lars Martin Jakt, Jarle T. Nordeide, Truls Moum, Steinar D. Johansen

**Affiliations:** 1 Genomics Group, Faculty of Biosciences and Aquaculture, Nord University, 8049 Bodø, Norway; 2 Research Laboratory and Department of Laboratory Medicine, Nordland Hospital, Bodø, Norway; 3 Department of Medical Biology, Faculty of Health Sciences, UiT–Arctic University of Norway, Tromsø, Norway; 4 Centre for Ecological and Evolutionary Syntheses (CEES), Department of Biosciences, University of Oslo, Oslo, Norway

**Keywords:** Atlantic cod, Mitogenome, *Gadus morhua*, Genomic resource, mtDNA, SNP

## Abstract

**Objective:**

The objective of this study was to analyse intraspecific sequence variation of Atlantic cod mitochondrial DNA, based on a comprehensive collection of completely sequenced mitochondrial genomes.

**Results:**

We determined the complete mitochondrial DNA sequence of 124 cod specimens from the eastern and western part of the species’ distribution range in the North Atlantic Ocean. All specimens harboured a unique mitochondrial DNA haplotype. Nine hundred and fifty-two polymorphic sites were identified, including 109 non-synonymous sites within protein coding regions. Eighteen variable sites were identified as indels, exclusively distributed in structural RNA genes and non-coding regions. Phylogeographic analyses based on 156 available cod mitochondrial genomes did not reveal a clear structure. There was a lack of mitochondrial genetic differentiation between two ecotypes of cod in the eastern North Atlantic, but eastern and western cod were differentiated and mitochondrial genome diversity was higher in the eastern than the western Atlantic, suggesting deviating population histories. The geographic distribution of mitochondrial genome variation seems to be governed by demographic processes and gene flow among ecotypes that are otherwise characterized by localized genomic divergence associated with chromosomal inversions.

**Electronic supplementary material:**

The online version of this article (10.1186/s13104-018-3506-3) contains supplementary material, which is available to authorized users.

## Introduction

The Atlantic cod (*Gadus morhua*) is one of the most important species for fisheries in the North Atlantic Ocean [1], and recently the nuclear genome was reported [2, 3]. The mitochondrial genome (mitogenome) is considered the second genome of the cell, and its gene content is conserved among most vertebrates [4]. The 16.7 kb Atlantic cod mitogenome encodes the standard set of 13 hydrophobic membrane proteins, 2 ribosomal RNAs (rRNAs), 22 transfer RNAs (tRNAs), as well as peptides and long non-coding RNAs, and is organized similarly to that of humans [5–7].

On average, the Atlantic cod mitogenome evolves about 14 times more rapidly at the nucleotide level than the nuclear genome [8], and mitochondrial sequence variation in cod was previously used to trace population structures and patterns of mitogenome evolution [8–12]. Árnason [9] investigated sequence variants of a 250-bp cytochrome b (CytB) gene fragment in 1278 Atlantic cod specimens throughout the distribution range, and identified trans-Atlantic haplotype clines with more diversity in northeastern and mid-Atlantic cod as compared to northwestern cod. Carr et al. [10, 12] reported on mitogenome variation based on 32 cod specimens and identified 298 single nucleotide polymorphic (SNP) sites. They found similar diversities in northwest and northeast Atlantic cod, but their sample from the northeast Atlantic consisted of six specimens only [10]. In the present study, we sequenced the complete mitogenome of 124 individuals, generating a mitochondrial sequence resource for future studies of Atlantic cod. We analysed relationships among the 156 cod mitogenomes currently available and compared mitogenome variation of the offshore migratory and stationary coastal cod ecotypes [11], both from the northeast Atlantic, and cod from the northwest Atlantic.

## Main text

### Methods

#### Tissue samples, nucleic acid extraction, PCR amplification, and plasmid cloning

Atlantic cod tissue samples were collected from the western (off Nova Scotia and Newfoundland) and eastern parts (off the British Isles, in the Baltic Sea, Irish Sea, North Sea, along the Norwegian coast and fjords, and in the Barents Sea) of the North Atlantic Ocean (Additional file 1: Table S1). DNA was extracted from fresh muscle tissue or ethanol preserved tissue (stored at − 20 °C) using the High Pure PCR Template Preparation kit (Roche) or the MasterPure™ Complete DNA and RNA Purification Kit (Epicentre^®^) according to the manufacturer’s protocols. Complete mitogenomes were PCR amplified in five overlapping fragments of approximately 4–4.5 kb in size using LaTaq polymerase (TAKARA BIO INC). The PCR products were purified using USB^®^ ExoSAP-IT^®^ reagent (Affymetrix). Agarose gel electrophoresis and gel extraction using Invitrogen™ PureLink^®^ Quick Gel Extraction Kit or Invitrogen™ PureLink^®^ PCR Purification Kit were performed according to the manufacturer’s protocols. PCR and sequencing primers used in this study have been described previously [13]. Plasmid cloning of the control region (CR) was performed by using Invitrogen™ TOPO^®^ TA Cloning^®^ Kit with One Shot^®^ TOP10 *E. coli* competent cells. Positive clones were cultivated and plasmid DNA was purified using Invitrogen™ PureLink^®^ Quick Plasmid Miniprep Kit.

#### Mitogenome sequencing and data analysis

The complete mitogenome sequences of 124 Atlantic cod specimens were determined, using Sanger, Illumina, and Roche 454 technologies (117, six, and one specimen, respectively). The latter, based on pyrosequencing, was reported previously [2]. The Illumina GAII sequencing was performed according to protocols in [14] using 2 × 108 bp paired end reads, library inserts of 550–575 bp, and 3.1–6.6 times (average 4.8 times) whole genome coverage (Norwegian Sequencing Centre—Oslo, Norway). Ninety-five mitogenomes were determined by Sanger sequencing provided by Eurofins MWG Operon (Germany). Two Sanger sequenced mitogenomes (NF1 and NC3) have been reported previously [8, 15]. The 20 remaining mitogenomes were sequenced in-house by Sanger technology directly on purified PCR products or plasmid DNA using the BigDye kit (Applied Biosystems). The complete 16,696-bp NC3 Atlantic cod mitogenome (HG514359) was used as a reference for assembly and mapping of mitogenome sequences and reads. Computer analyses of Sanger-generated mitogenomes were performed using DNASTAR^®^ Lasergene software. For mitogenome sequences generated by Roche 454 and Illumina platforms, reference mappings were performed on the CLC Genomics Workbench (QIAGEN^®^).

A total of 156 available mitogenomes were used to calculate population genetic parameters and reconstruct molecular relationships among Atlantic cod specimens. The CR, tRNA-Phe, and half of the tRNA-Pro sequence were excluded from these comparisons, as these sequences were not available for the 32 specimens previously reported [10]. Population genetic statistics and measures of genetic differentiation were estimated for the following three subsets of specimens, defined by their geographic origin and ecotype: cod from the northwest Atlantic (NW; N = 32), cod from the north east Atlantic of the coastal stationary ecotype (NC; N = 25), and Arctic cod from the Barents Sea of the migratory ecotype (NA; N = 97) (Additional file 1: Table S1). Two specimens from the Baltic Sea were excluded from these analyses, since differentiation from NC due to vicariance is likely. Nucleotide sequence alignments were generated using T-coffee v/9 software [16] with manual refinements. The tree-building method of maximum likelihood (ML) in MEGA version 6 [17] was used to reconstruct molecular relationships. The ML trees were built from best-fit models of nucleotide evolution generated by MEGA6 [Bayesian information criterion calculations resulted in TN93+I+G as best-fit model]. The topologies of the ML trees were evaluated by bootstrap analyses (2000 replications). We analysed nucleotide diversity indices, Tajima’s D statistic, and genetic differentiation indices *F*_ST_ and Da (the average number of net nucleotide substitutions), as implemented in the DnaSP version 6 software [18].

### Results

#### Sequence variation among Atlantic cod mitogenomes

Complete mitogenome sequences of approximately 16.7 kb were obtained for 124 cod specimens sampled in the western and eastern parts of the North Atlantic Ocean. Mitochondrial sequence variation was initially assessed by considering nucleotide variants of the CytB gene fragment (250 bp) previously reported for 1278 Atlantic cod specimens throughout the species’ range [9]. Eleven haplotypes from that study, including all main haplotypes (A, C, D, E and G) were identified among the 124 specimens, as well as 12 other singleton haplotypes (Additional file 2: Table S2).

**Fig. 1:**
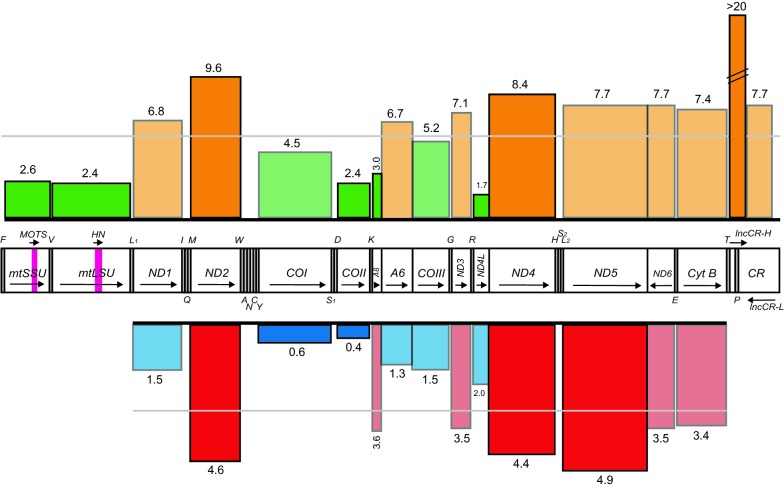
Gene content and variability of Atlantic cod mitogenomes. Mitogenome presented as a linear map of the circular mtDNA. Single nucleotide polymorphisms (SNPs per 100 bp) in gene regions detected among the 124 completely sequenced mitogenomes are indicated above the gene map. Grey horizontal line denotes the average number of SNP (5.5) per 100 bp that include all genes. Genes above and below this average are shown as orange and green bars, respectively. Amino acid substitutions in mitochondrial proteins are presented below the gene map. Grey horizontal line denotes the average number of substitutions (2.9) per 100 amino acids that include all proteins. Proteins above and below this average are shown as red and blue bars, respectively. *mtSSU* and *mtLSU* mitochondrial small- and large-subunit ribosomal RNA genes, *ND1-6* NADH dehydrogenase subunit 1–6, *COI-III* cytochrome c oxidase subunit I to III, *A6* and *A8* ATPase subunit 6 and 8, *Cyt B* cytochrome b, *MOTS* putative MOTS-c peptide, *HN* putative humanin peptide, *lncCR-H* and *lncCR-L* long non-coding RNAs coded by the control region (CR). See [5–7] for more details about mitochondrial gene products. tRNA genes are indicated by the standard one-letter symbols for amino acids. All genes are H-strand encoded, except Q, A, N, C, Y, S_1_, E, P, ND6, and lncCR-L (L-strand encoded)

The 124 mitogenome sequences were unambiguously aligned using the Norwegian costal NC3 (HG514359) [8] as an Atlantic cod reference, resulting in an alignment of 16,551 positions. The total number of polymorphic sites was 952 (5.7% of mitogenome positions), and these were distributed across the two rRNA genes, all 13 protein coding genes, 18 of the 22 tRNA genes, and major non-coding regions (TP-spacer and CR) (Fig. 1). Only 18 variable sites (1.9%) were identified as indels (6 in structural RNA genes and 12 in non-coding regions). Protein coding genes contained 756 (79.4%) substitutions, of which 109 were non-synonymous (14.4%), resulting in amino acid changes in all 13 proteins (Additional file 3: Table S3).

Key features of mitogenome sequence variation are summarized in Additional file 4: Table S4, and several features are noted. Protein coding genes have 2.3 times more SNPs per nucleotide position than RNA genes. This reflect high sequence conservation in RNA genes at the species level. An interesting observation is that variable sites in the mitochondrial small subunit rRNA gene are mainly clustered within the 3′M structural domain (Additional file 5: Figure S1). A similar feature was not noted in the mitochondrial large subunit rRNA (Additional file 6: Figure S2). The cytochrome oxidase (CO) genes and proteins (Complex IV) are generally more conserved than the NADH dehydrogenase (ND) genes and proteins (Complex I). Here, the ND2 gene contains 7.2 times more polymporphic sites per position than, e.g. COII or the RNA genes. The ND2, ND4, ND5, and CytB genes contain the highest density of polymorphic sites.

#### Mitogenome diversity, intraspecific relationships and genetic differentiation

**Fig. 2:**
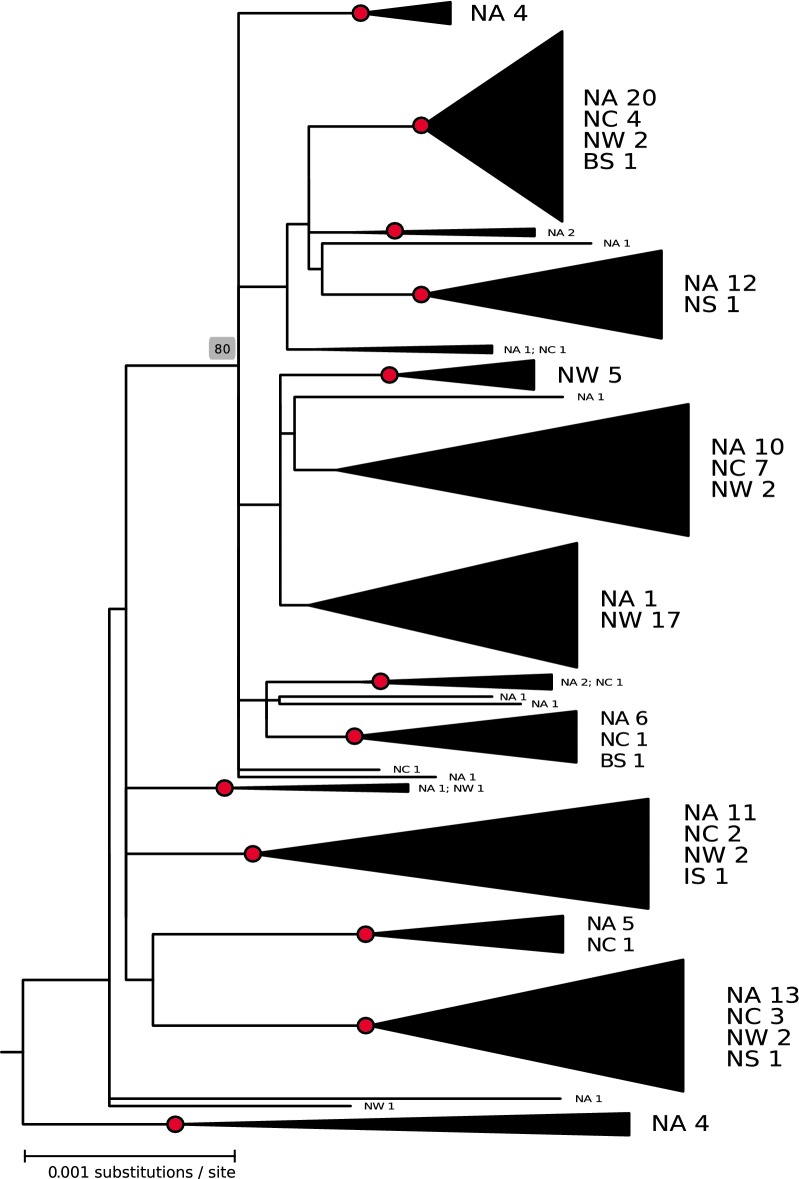
Mitogenome relationships in Atlantic cod. Maximum likelihood (ML) phylogenetic tree based on complete mitogenome haplotype sequences (15,592 common nucleotide positions) of 156 Atlantic cod specimens. *Theragra finnmarchica* (Norwegian Pollock; AM489718) mitogenome was used as an outgroup in tree construction. Bootstrap values (%) from 2000 replicates, all over 70%, are shown at branches. Red filled circles indicate highly significant branch points of bootstrap values above 80% in ML analysis. Closely related haplotype clades are collapsed (bootstrap values above 60%). *NA* Northeast Arctic cod, *NC* Norwegian costal cod, *NW* Northwest cod, *BS* Baltic Sea cod, *IS* Irish Sea cod, *NS* North Sea cod

The alignment of 156 available cod mitogenomes resulted in 15,592 common sites following the exclusion of alignment gaps. There were 1002 polymorphic sites, and 1034 substitutions in total among the mitogenome sequences. The nucleotide diversity index was slightly lower for cod in the northwest Atlantic (0.203%) compared to stationary and migratory cod in the northeast Atlantic (0.291 and 0.285%, respectively; Table 1). Tajima’s D statistic was significantly negative for all population subsets, rejecting the null hypothesis of stable populations with no selection. A representative maximum likelihood (ML) tree is shown in Fig. 2. Twelve clades were supported in > 80% of bootstrap replications, but there was not a clear geographic structuring of clades. However, while some clades were dominated by NA and NC cod individuals, others harboured mainly NW cod (Fig. 2). Measures of pairwise genetic differentiation were negative (interpreted as nil) between NA and NC cod in the northeast Atlantic, while NW cod were differentiated from both NC and NA cod (F_ST_ of approximately 0.06 and 0.09, respectively; Additional file 7: Table S5).

**Table 1:** Population genetic parameters of Atlantic cod based on the alignment of nearly complete mitochondrial DNA sequences

	π %	S	η	k	TD
NW	0.203	306	308	31.60	− 2.26**
NC	0.291	339	344	45.41	− 2.01*
NA	0.285	724	743	44.47	− 2.36**
Total (N = 156)	0.276	1002	1034	42.99	− 2.51***

Sites with alignment gaps were excluded from the alignment of 16,551 positions in all subsets resulting in 15,592 common sites. NW, cod from the north west Atlantic (N = 32); NC, cod from the north east Atlantic of the coastal stationary ecotype (N = 25); NA, Arctic cod from the Barents Sea of the migratory ecotype (N = 97); π %, percent nucleotide diversity; S, number of segregating sites; η, total number of substitutions; k, average number of pairwise nucleotide differences; TD, Tajima’s D statistic. * P < 0.05; ** P < 0.01; *** P < 0.001

### Discussion

Here we provide a comprehensive SNP map of the Atlantic cod mitochondrial genome based on 124 completely sequenced mtDNAs. The 952 variable sites identified among 124 specimens were not evenly distributed throughout the mitogenome. Structural RNA genes have a significantly lower density of overall SNPs per site and variable sites per position compared to protein coding genes. Furthermore, the ND2 gene and the COII gene were the least and most conserved, respectively, among the protein coding genes. This feature was also observed at the protein level. Thus, the Atlantic cod mitogenome follow a similar pattern of conservation as seen for, e.g. zebrafish [19] or humans [20, 21].

One hundred and twenty-four cod individuals harboured substantial sequence variation in their mitogenomes, including 349 phylogenetically informative parsimony sites. Phylogenetic analysis of 156 available mitogenomes identified ten haplotype clusters supported by high bootstrap values, but little phylogeographic structuring. Mitogenome evolution in cod seems to be nearly neutral [8, 12], suggesting that the significantly negative Tajima’s D statistic mainly signifies recent demographic change, rather than selection. The differentiation of certain cod populations into so-called ecotypes defined by migratory and stationary behaviour, most notably NC and NA cod in the northeastern Atlantic, has long been a conundrum [11]. Recently, it was shown that these ecotypes are associated with genomic islands of differentiation, inferred to reside within chromosomal inversions in at least four linkage groups [22, 23]. It is conceivable that such genomic regions could preclude recombination and break-up of co-adapted genes within them, and thus make it possible for locally adapted ecotypes to persist in the face of continued gene flow. Similar chromosomal inversions, suggesting a common ancestry, were subsequently found to contribute to ecotype divergence in the western Atlantic as well [24]. The mitogenome data indicate some differentiation between western and eastern cod, but a lack of differentiation between NC and NA cod. This would be consistent with isolation by distance and some gene flow between ecotypes in their mitochondrial genes and neutrally evolving parts of the nuclear genome. Thus, the geographic structuring of mitogenome variation in cod seems to be governed mainly by demographic and stochastic processes in a species with high fecundity and variance in offspring number, much in line with Árnason’s conclusions based on CytB sequences [9].

### Conclusion

Our study provides a mitochondrial genome resource obtained from Atlantic cod tissue samples collected at site of fisheries in the North Atlantic Ocean. Phylogeographic analyses based on 156 mitochondrial genomes did not reveal a clear structure, but eastern and western cod were differentiated. Mitochondrial genome diversity was higher in the eastern than the western Atlantic, suggesting deviating population histories.

## Limitations

The SNP map of the Atlantic cod mitochondrial genome consisted of 952 polymorphic sites among the 124 specimens studied here, and 1002 polymorphic sites among 156 available mitogenomes from the western and eastern parts of the Atlantic Ocean. A more exhaustive SNP map of the cod mitogenome would most certainly require a substantial increase in the number of mitogenomes collected from the vast distribution range of the species.

## Additional files

**Additional file 1: Table S1.** Geographical distribution of completely sequenced Atlantic cod mitogenomes.

**Additional file 2: Table S2.** Mitochondrial CytB haplotypes generated from 124 complete Atlantic cod mitogenomes.

**Additional file 3: Table S3.** Non-synonymous substitutions in 124 Atlantic cod mitogenomes.

**Additional file 4: Table S4.** Key features of SNPs in 124 complete Atlantic cod mitogenomes.

**Additional file 5: Figure S1.** Complete secondary structure diagram of Atlantic cod mitochondrial small subunit rRNA. Variable positions among the 124 complete Atlantic cod mitogenomes are indicated, as well as frequency (%) and variable sites (red boxes).

**Additional file 6: Figure S2.** Complete secondary structure diagram of Atlantic cod mitochondrial large subunit rRNA. (A) Domains O and II. (B) Domains III and IV. (C) Domains V and VI. Variable positions among the 124 complete Atlantic cod mitogenomes are indicated, as well as frequency (%) and variable sites (red boxes).

**Additional file 7: Table S5.** Genetic differentiation among population subsets defined by geography and ecotype based on nearly complete mitochondrial DNA sequences.

## Abbreviations

CO: cytochrome oxidase
CR: control region
CytB: cytochrome b
LSU: large subunit
ML: maximum likelighood
mtDNA: mitochondrial DNA
ND: NADH dehydrogenase
SNP: single nucleotide polymorphism
SSU: small subunit
