# Supplementary material for: Mitochondrial genome variation of Atlantic cod
Source: BMC Res Notes. 2018 Jun 19;11:397. doi: 10.1186/s13104-018-3506-3 (PMC6009815; doi:10.1186/s13104-018-3506-3)
Supplement: Supplementary file 1 — Additional file 1: Table S1. Geographical distribution of completely sequenced Atlantic cod mitogenomes. [file 13104_2018_3506_MOESM1_ESM.pdf]

Additional file 1: Table S1.
Geographical distribution of completely sequenced Atlantic cod mitogenomes

Management unit/ Stock/ Location	Number of specimens	Reference
North East Arctic cod NA	97	PRJEB23234/ERP104973
Norwegian coastal cod NC	21	HG514359; PRJEB23234/ERP104973; EU877736–EU877741
North West Atlantic cod NW	32	AM489716; PRJEB23234/ERP104973; EU877710–EU877735
Other locations:		
Baltic Sea cod BS	2	PRJEB23234/ERP104973
North Sea cod NS	3	PRJEB23234/ERP104973
Irish Sea cod IS	1	PRJEB23234/ERP104973
