# Supplementary material for: Mitochondrial genome variation of Atlantic cod
Source: BMC Res Notes. 2018 Jun 19;11:397. doi: 10.1186/s13104-018-3506-3 (PMC6009815; doi:10.1186/s13104-018-3506-3)
Supplement: Supplementary file 2 — Additional file 2: Table S2. Mitochondrial CytB haplotypes generated from 124 complete Atlantic cod mitogenomes. [file 13104_2018_3506_MOESM2_ESM.pdf]

Additional file 2: Table S2. Mitochondrial CytB haplotypes generated from 124 complete Atlantic cod mitogenomes.

Haplo-	4	4	4	4	4	4	4	4	4	4	5	5	5	5	5	5	5	5	5	5	5	5	5	5	5	5	5	6	6	6	6	6	6	6	6	6	6	6	6	6	6	7	
type[1]	6	6	6	6	7	8	8	8	9	9	0	0	1	1	2	2	3	4	4	4	4	5	6	6	8	8	9	0	3	3	4	4	5	5	6	7	7	8	9	9	9	0	N
	3	6	7	8	5	1	7	8	0	6	2	8	4	7	2	3	5	1	2	4	7	6	2	5	6	9	5	1	1	7	3	9	5	8	4	3	6	5	1	5	7	6	
A	C	A	A	T	A	A	A	C	A	A	A	T	A	C	A	G	A	A	G	C	C	T	A	C	T	A	T	T	G	T	A	C	T	T	T	A	G	C	G	G	C	T	39
E	.	.	.	.	.	.	.	.	.	.	.	C	.	.	.	.	.	.	.	.	.	.	.	.	.	.	.	.	.	.	.	.	.	.	.	.	.	.	.	.	.	.	35
D	.	.	.	.	.	.	.	.	.	.	.	.	.	.	.	A	.	.	.	.	.	.	.	.	.	.	.	.	.	.	.	.	.	.	.	.	.	T	.	.	.	.	16
G	.	.	.	.	.	.	.	.	.	.	.	.	.	.	.	.	.	.	.	.	.	.	.	.	.	.	.	.	.	.	.	.	.	.	.	.	.	.	T	.	.	.	10
C	.	.	.	.	.	.	.	.	.	.	.	.	.	.	.	.	.	.	.	.	.	.	.	.	.	.	.	.	.	.	.	.	.	.	.	.	.	T	.	.	.	.	3
NI	.	.	.	.	.	.	.	.	.	.	.	.	.	.	.	.	.	.	.	.	.	.	.	.	.	.	.	.	A	.	.	.	.	.	.	.	.	.	T	.	.	.	3
XI	.	.	.	.	.	.	.	.	G	.	.	C	.	.	.	.	.	.	.	.	.	.	.	.	.	.	.	.	.	.	.	.	.	.	.	.	.	.	.	.	.	.	2
	.	.	.	.	.	.	.	.	.	.	.	.	.	.	.	.	.	.	.	.	.	.	.	.	.	T	.	.	.	.	.	.	.	.	.	.	.	.	.	.	.	.	1
	.	.	.	.	.	.	.	.	.	.	.	.	.	.	.	.	.	.	.	.	.	.	.	.	.	.	.	.	.	.	.	.	.	.	.	G	.	T	.	.	.	.	1
	.	.	.	.	.	.	.	.	.	.	.	.	.	.	.	.	.	.	.	.	.	.	.	.	.	.	.	.	.	.	.	.	.	.	.	.	.	T	.	A	.	.	1
	.	.	.	.	.	.	.	.	.	.	.	.	.	.	.	.	.	.	.	.	.	.	.	.	.	.	.	.	.	.	.	.	C	.	.	.	.	.	.	.	.	.	1
P	.	.	.	.	.	.	.	.	.	.	.	.	.	.	.	.	.	.	.	.	.	.	.	.	.	.	.	.	.	C	.	.	.	.	.	.	.	.	.	.	.	.	1
S	.	.	.	.	.	.	.	.	.	.	.	.	.	.	.	.	.	.	.	.	.	.	.	.	.	.	.	.	.	.	.	T	.	.	.	.	.	.	.	.	.	.	1
DI	.	.	.	.	.	.	.	.	.	.	.	.	.	.	.	A	.	.	.	.	.	.	.	.	.	.	.	.	.	.	.	.	.	.	.	.	.	T	A	.	.	.	1
	.	.	.	.	.	.	.	.	.	.	.	.	.	.	.	.	.	G	.	.	.	.	.	.	.	.	.	.	.	.	.	.	.	.	.	.	.	T	.	.	.	.	1
	.	.	.	.	.	.	.	.	.	.	.	.	.	.	.	.	.	.	.	.	.	.	G	.	.	.	.	.	.	.	.	.	.	.	.	.	.	.	T	.	.	.	1
	.	.	.	.	.	.	.	.	.	.	.	.	.	.	.	.	G	.	.	.	.	.	.	.	.	.	.	.	.	.	.	.	.	.	.	.	.	.	T	.	.	.	1
TI	.	.	.	.	.	.	.	.	.	.	G	.	.	.	.	.	.	.	.	.	.	.	.	.	.	.	.	.	.	.	.	.	.	.	.	.	.	.	T	.	.	.	1
	.	.	.	.	.	.	.	.	G	.	.	C	.	.	.	.	.	.	A	.	.	.	.	.	.	.	.	.	.	.	.	.	.	.	.	.	.	.	.	.	.	.	1
	.	.	.	.	.	.	.	.	.	.	G	C	.	.	.	.	.	.	.	.	.	.	.	.	.	.	.	.	.	.	.	.	.	.	.	.	.	.	.	.	.	.	1
	.	.	.	.	.	.	.	.	.	.	.	C	.	.	.	.	G	.	.	.	.	.	.	.	.	.	.	.	.	.	.	.	.	.	.	.	.	.	.	.	.	.	1
	.	.	.	.	.	.	.	.	.	.	.	C	.	.	.	.	.	.	.	.	.	C	.	.	.	.	.	.	.	.	.	.	.	.	.	.	.	.	.	.	.	.	1
	.	.	.	.	.	.	.	.	.	.	.	C	.	.	.	.	.	.	.	.	.	.	.	.	.	.	.	.	.	.	.	.	.	.	.	G	.	.	.	.	.	.	1

Note: 1 Position 14463 – 14706, positions are according to the reference sequence NC3 (HG514359)
