# Supplementary material for: Mitochondrial genome variation of Atlantic cod
Source: BMC Res Notes. 2018 Jun 19;11:397. doi: 10.1186/s13104-018-3506-3 (PMC6009815; doi:10.1186/s13104-018-3506-3)
Supplement: Supplementary file 3 — Additional file 3: Table S3. Non-synonymous substitutions in 124 Atlantic cod mitogenomes. [file 13104_2018_3506_MOESM3_ESM.pdf]

Additional file 3: Table S3 Non-synonymous substitution in 124 Atlantic cod mitogenomes.

[1]Position	[2]Gene	Codon	Amino acid	Position	Gene	Codon	Amino acid
3154	ND1	ATT → ACT	I → T	5583	COI	CTT → CCT	L → P
3177	ND1	AGT → GGT	S → G	6662	COI	GAT → AAT	D → N
3607	ND1	GAA → GGA	E → G	7278	COII	GTC → ATC	V → I
3646	ND1	TTA → TCA	L → S	7936	ATPase8	GCC → ACC	A → T
3651	ND1	ATG → GTG	M → V	7955	ATPase8	TTC → TCC	F → S
4039	ND2	ATC → ACC	I → T	8316	ATPase6	CTC → TTC	L → F
4138	ND2	ATC → ACC	I → T	8637	ATPase6	GCA → ACA	A → T
4236	ND2	AGC → GGC	S → G	8638	ATPase6	GCA → GTA	A → V
4293	ND2	TTC → CTC	F → L	9050	COIII	CTC → TTC	L → F
4462	ND2	AAC → AGC	N → S	9113	COIII	ACA → GCA	T → A
4491	ND2	ACC → GCC	T → A	9254	COIII	ACA → GCA	T → A
4545	ND2	ATC → GTC	I → V	9442	COIII	TCC → CCC	S → P
4681	ND2	TCT → TGT	S → C	9638	ND3	CTT → TTT	L → F
4754	ND2	ATT → ATG	I → M	9650	ND3	GCT → ACT	A → T
4834	ND2	GAT → GGT	D → G	9662	ND3	ATT → GTT	I → V
4893	ND2	CTC → ATC	L → I	9869	ND3	ACC → GCC	T → A
4974	ND2	GCA → ACA	A → T	10088	ND4L	TTA → TTC	L → F
4975	ND2	GCA → GTA	A → V	10312	ND4L	AAT → AGT	N → S
4996	ND2	ATT → ACT	I → T	[3]10322	ND4	ATG → GTG	M → V
5014	ND2	GCT → GTT	A → V	10370	ND4	TTA → ATA	L → M
5041	ND2	ACC → ATC	T → I	10440	ND4	ACC → ATC	T → I
5453	COI	ACC → TCC	T → S	10484	ND4	TCA → CCA	S → P

Position	Gene	Codon	Amino acid	Position	Gene	Codon	Amino acid
10591	ND4	ATA → ATT	M → I	12721	ND5	ATG → ATC	M → N
10593	ND4	AAC → AGC	N → S	12737	ND5	AAT → GAT	N → V
10632	ND4	CAG → CGG	Q → R	12906	ND5	GCC → GTC	A → V
10833	ND4	AAC → AGC	N → S	12908	ND5	TTT→ TCT	F → S
10859	ND4	ATC → CTC	I → L	13028	ND5	GCC → ACC	A → T
10889	ND4	CCC → TCC	P → S	13113	ND5	GCA → GTA	A → V
10898	ND4	AGT → GGT	S → G	13168	ND5	ACC → GCC	T → A
11247	ND4	GGA → GAA	G → E	13208	ND5	GTT → ATT	V → I
11264	ND4	ATT → GTT	I → V	13247	ND5	CCC → TCC	P → S
11405	ND4	ATT → GTT	I → V	13274	ND5	CCA → TCA	P → S
11466	ND4	ATT → ACT	I → T	13382	ND5	GCA → ACA	A → T
11484	ND4	AAC → AGC	N → S	13382	ND5	GCA → CCA	A → P
11504	ND4	ATT → GTT	I → V	13383	ND5	GCA → GTA	A → V
11597	ND4	GCC → ACC	A → T	13415	ND5	ATT → GTT	I → V
11678	ND4	GCC → ACC	A → T	13436	ND5	CTT → TTT	L → F
11691	ND4	GGC → GCC	G → A	13458	ND5	AGC → AAC	S → N
12024	ND5	GCC → GTC	A → V	13472	ND5	GTT → ATT	V → I
12074	ND5	GCT → ACT	A → T	13476	ND5	ACA→ ATA	T → M
12096	ND5	AGC → AAC	S → N	13520	ND5	GTC → ATC	V → I
12110	ND5	GCT → ACT	A → T	13601	ND5	ACA → GCA	T → A
12266	ND5	CTC → TTC	L → F	13640	ND5	GCT → ACT	A → T
12489	ND5	GCA → GTA	A → V	13673	ND5	ATA → GTA	M → V
12548	ND5	AAT → GAT	N → D	13739	ND5	ATT → GTT	I → V

Position	Gene	Codon	Amino acid	Position	Gene	Codon	Amino acid
13969	ND6	GCA → GTA	A → V	14828	Cyt b	CAA → CGA	Q → R
13975	ND6	GTG → GCG	V → A	14843	Cyt b	GGT → GAT	G → D
13976	ND6	GTG → ATG	V → M	14980	Cyt b	TCA → CCA	S → P
14144	ND6	TTA → GTA	L → V	15004	Cyt b	CAC → AAC	H → N
14177	ND6	CTG → GTG	L → V	15140	Cyt b	CCT → CAT	P → H
14251	ND6	ACT → AGT	T → S	15322	Cyt b	GTC → ATC	V → I
14542	Cyt b	GTC → ATC	V → I	15382	Cyt b	TTC → CTC	F → L
14589	Cyt b	ATA → ATT	M → I	15409	Cyt b	GTA → ATA	V → M
14695	Cyt b	GTC → ATC	V → I	15457	Cyt b	ACT → GCT	T → A
14719	Cyt b	GCC → ACC	A → T				

Note: 1 Positions are according to the reference sequence NC3 (HG514359)

2 ND1 - NADH dehydrogenase subunit 1; ND2 - NADH dehydrogenase subunit 2; ND3 - NADH dehydrogenase subunit 3; ND4 – NADH dehydrogenase subunit 4; ND4L - NADH dehydrogenase subunit 4L; ND5 - NADH dehydrogenase subunit 5; ND6 – NADH dehydrogenase subunit 6; COI - cytochrome c oxidase subunit I; COII - cytochrome c oxidase subunit II; COIII - cytochrome c oxidase subunit III; ATPase8 - ATP synthase 8; ATPase6 -ATP synthase 6 ; Cyt b - Cytochrome B.

3 Start codon of ND4
