# Supplementary material for: Mitochondrial genome variation of Atlantic cod
Source: BMC Res Notes. 2018 Jun 19;11:397. doi: 10.1186/s13104-018-3506-3 (PMC6009815; doi:10.1186/s13104-018-3506-3)
Supplement: Supplementary file 4 — Additional file 4: Table S4. Key features of SNPs in 124 complete Atlantic cod mitogenomes. [file 13104_2018_3506_MOESM4_ESM.pdf]

Additional file 4: Table S4. Key features of SNPs in 124 complete Atlantic cod mitogenomes

Gene	SNPs	SNPs /100bp	Singleton Sites[1]
ND1	66	6.8	38
ND2	100	9.6	55
ND3	25	7.1	15
ND4L	5	1.7	4
ND4	117	8.4	71
ND5	141	7.7	79
ND6	40	7.7	25
COI	69	4.5	49
COII	17	2.4	13
COIII	41	5.2	26
ATPase6	46	6.7	36
ATPase8	5	3.0	5
Cyt b	84	7.4	48
SSU	25	2.6	18
LSU	52	3.1	45
tRNAs	41	2.7	32
Protein genes	756	6.6	464
RNA genes	118	2.8	95

1 Non-informative site. Nucleotide variant that appears only once in the data set.
