# Supplementary figures and images for: Mitochondrial genome variation of Atlantic cod
Source: BMC Res Notes. 2018 Jun 19;11:397. doi: 10.1186/s13104-018-3506-3 (PMC6009815; doi:10.1186/s13104-018-3506-3)

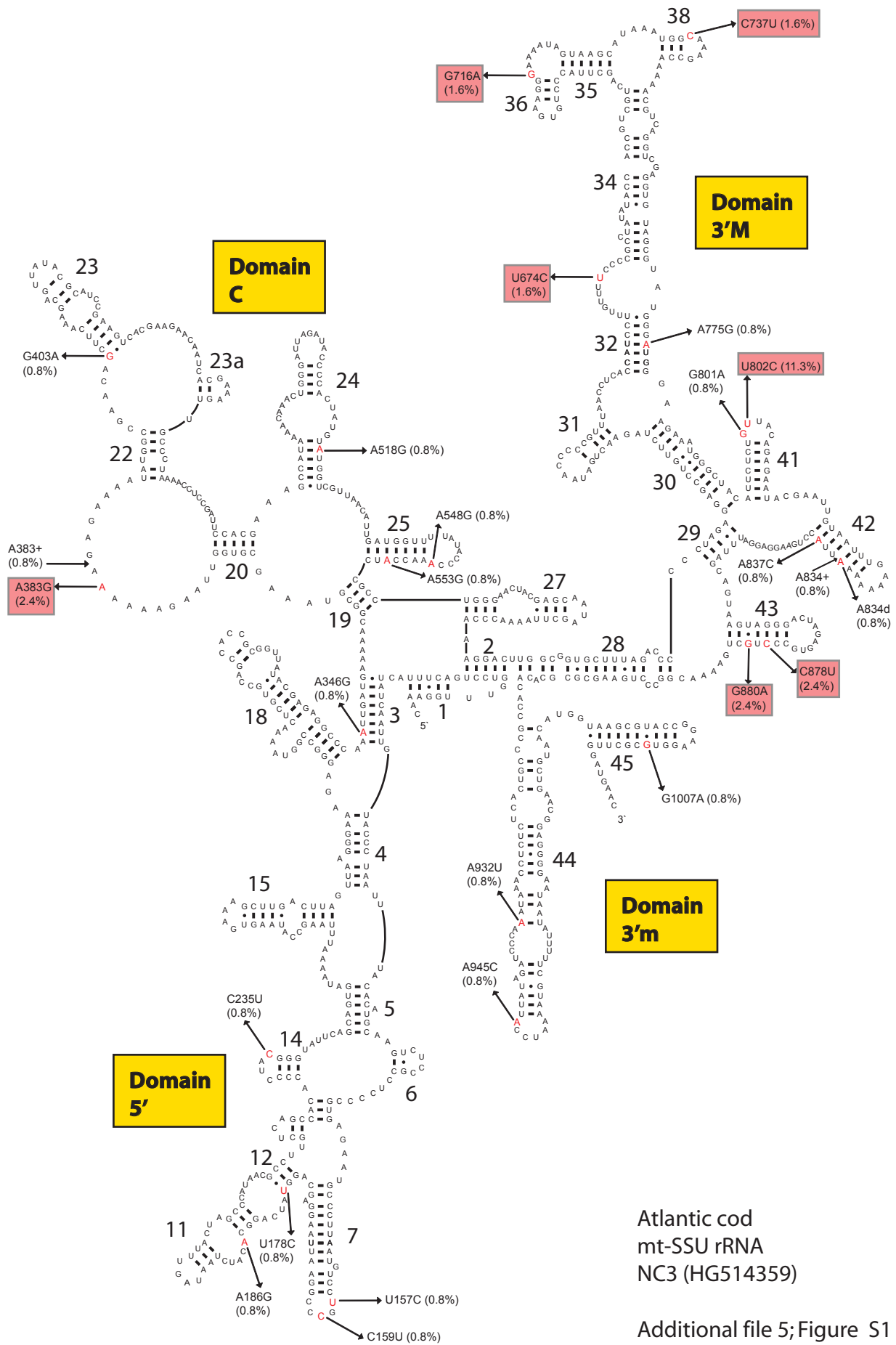

Supplement: Supplementary file 5 — Additional file 5: Figure S1. Complete secondary structure diagram of Atlantic cod mitochondrial small subunit rRNA. Variable positions among the 124 complete Atlantic cod mitogenomes are indicated, as well as frequency (%) and variable sites (red boxes). [file 13104_2018_3506_MOESM5_ESM.pdf]
