# Supplementary material for: Mitochondrial genome variation of Atlantic cod
Source: BMC Res Notes. 2018 Jun 19;11:397. doi: 10.1186/s13104-018-3506-3 (PMC6009815; doi:10.1186/s13104-018-3506-3)
Supplement: Supplementary file 6 — Additional file 6: Figure S2. Complete secondary structure diagram of Atlantic cod mitochondrial large subunit rRNA. (A) Domains O and II. (B) Domains III and IV. (C) Domains V and VI. Variable positions among the 124 complete Atlantic cod mitogenomes are indicated, as well as frequency (%) and variable sites (red boxes). [file 13104_2018_3506_MOESM6_ESM.pdf]

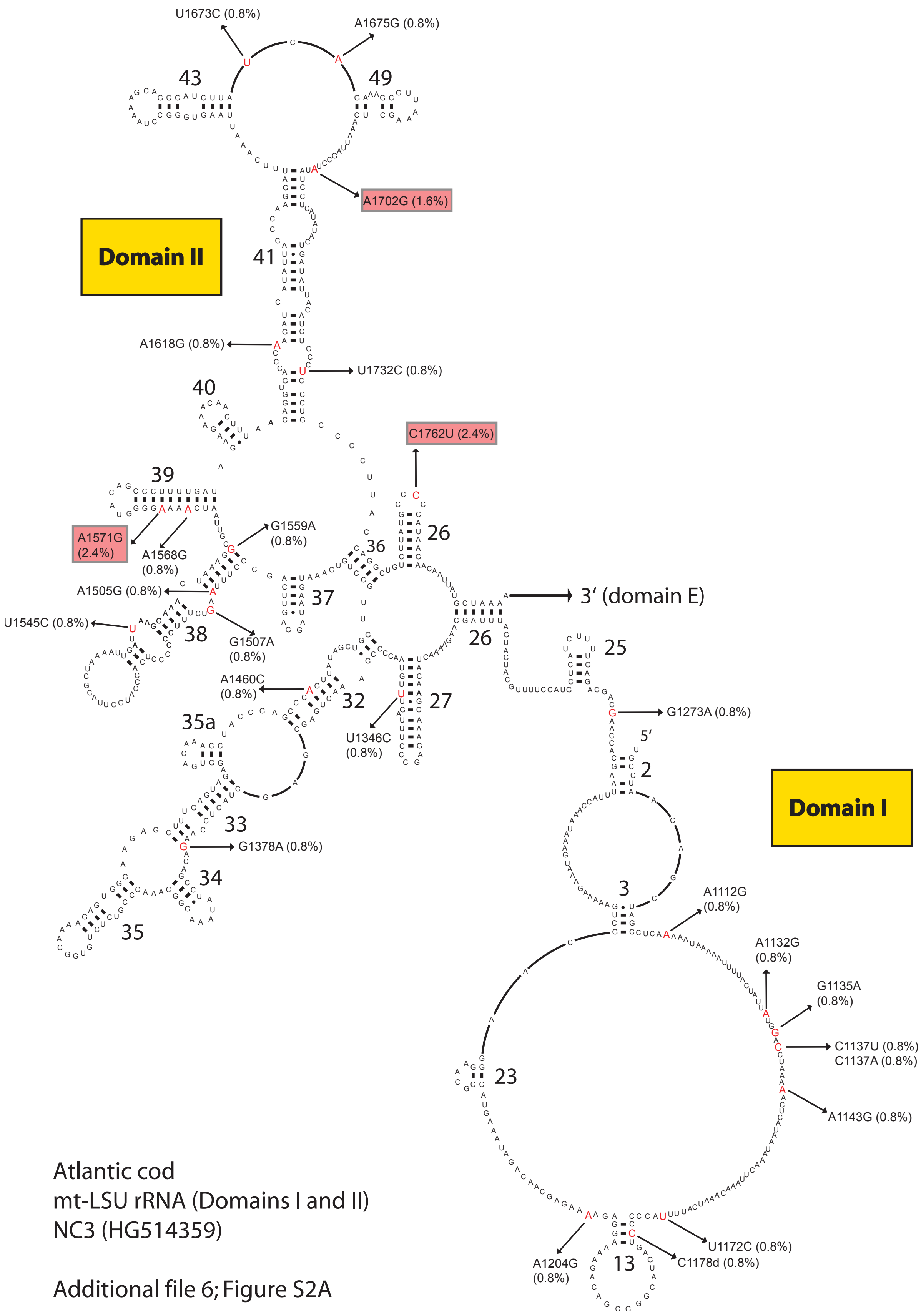

Atlantic cod
mt-LSU rRNA (Domains I and II)
NC3 (HG514359)

Additional file 6; Figure S2A

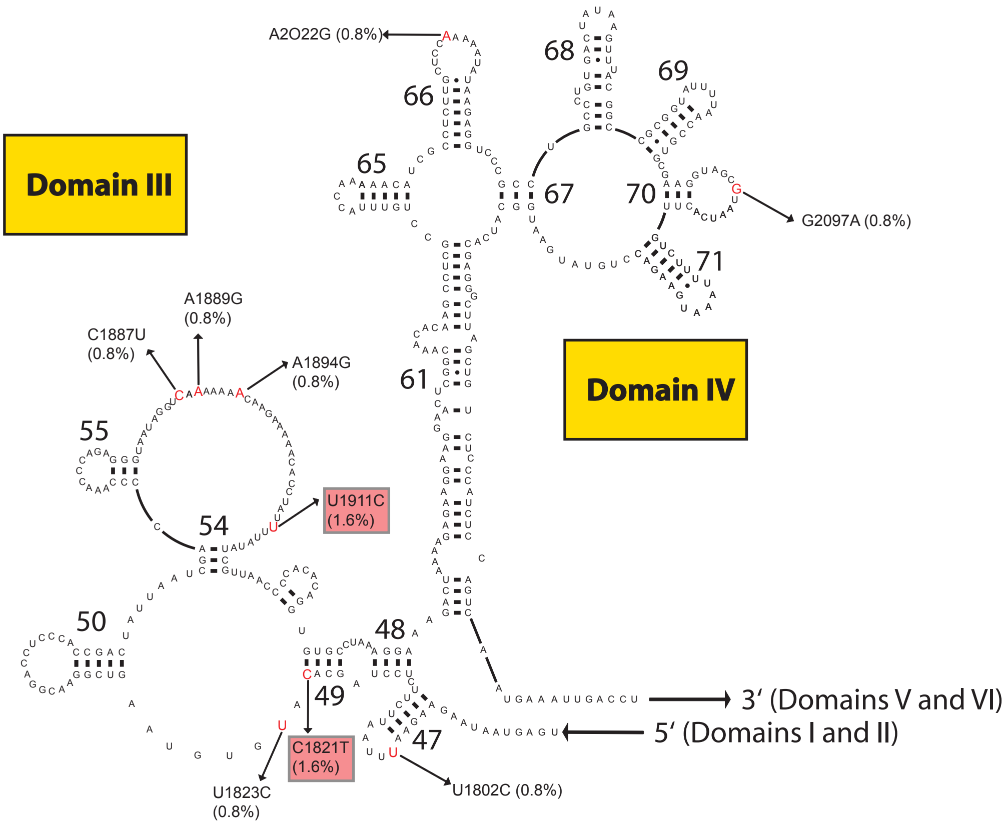

Atlantic cod
mt-LSU rRNA (Domains III and IV)
NC3 (HG514359)

Additional file 6: Figure S2B

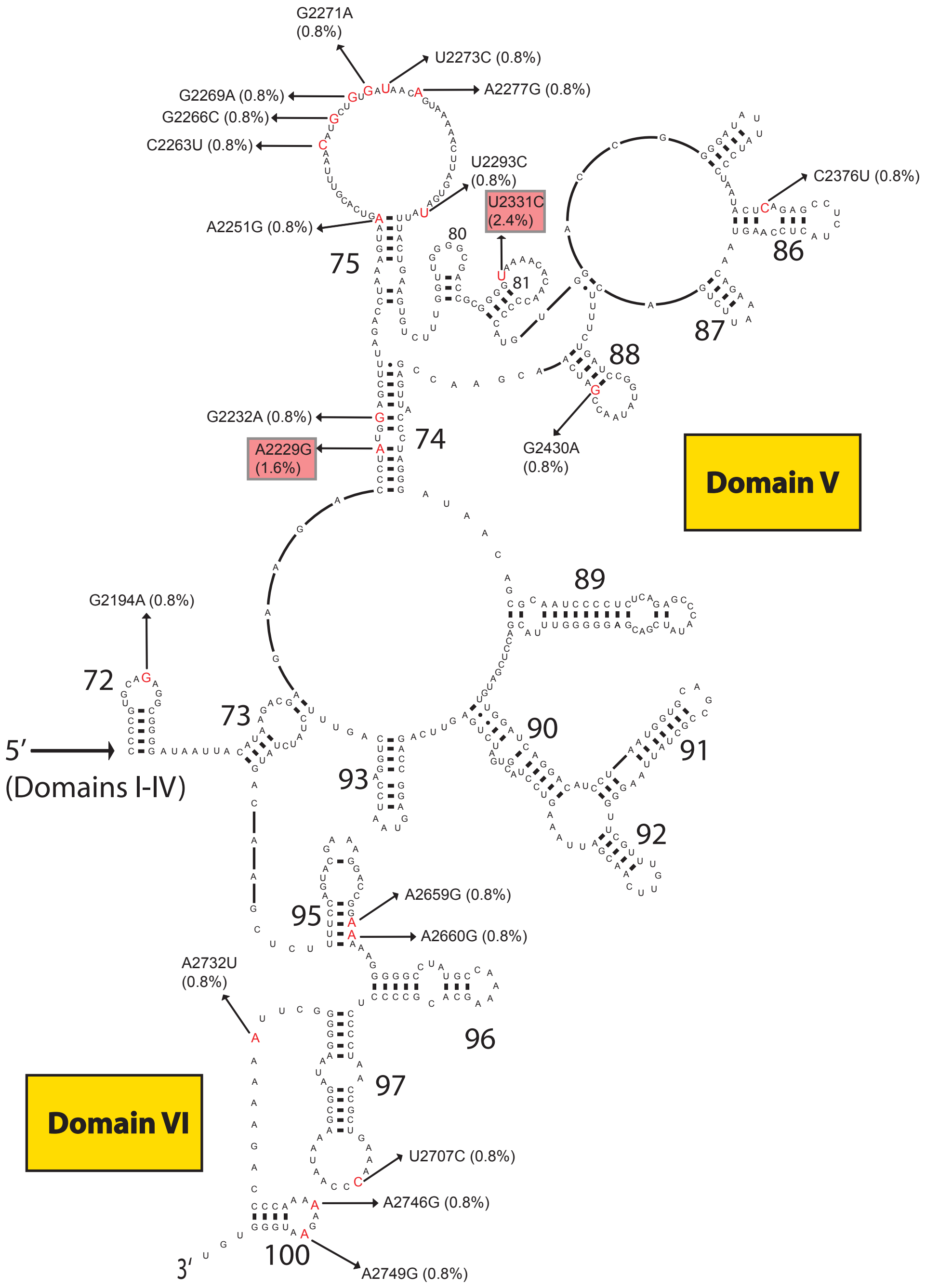

Atlantic cod
mt-LSU rRNA (Domains V and VI)
NC3 (HG514359)

Additional file 6: Figure S2C
