# Supplementary material for: Mitochondrial genome variation of Atlantic cod
Source: BMC Res Notes. 2018 Jun 19;11:397. doi: 10.1186/s13104-018-3506-3 (PMC6009815; doi:10.1186/s13104-018-3506-3)
Supplement: Supplementary file 7 — Additional file 7: Table S5. Genetic differentiation among population subsets defined by geography and ecotype based on nearly complete mitochondrial DNA sequences. [file 13104_2018_3506_MOESM7_ESM.pdf]

Additional file 7: Table S5. Genetic differentiation among population subsets defined by geography and ecotype based on nearly complete mitochondrial DNA sequences

Comparison	F_{ST}	Dxy	Da
NW vs NC	0.05851	0.00262	0.00015
NW vs NA	0.08832	0.00268	0.00024
NC vs NA	-0.00499	0.00287	-0.00001

Sites with alignment gaps were excluded from the alignment of 16 551 positions in all subsets resulting in 15 592 common sites. Population subsets NW, NC, and NA: see Table 2 and main text. F_{ST}, fixation index; Dxy, average number of nucleotide substitutions between subsets; Da, average number of net nucleotide substitutions between subsets.
